# Prognostic Significance of Serum Proangiogenic Molecules in Patients with *De Novo* Non-Hodgkin Lymphomas

**DOI:** 10.1100/2012/215231

**Published:** 2012-04-26

**Authors:** Pairaya Rujirojindakul, Arnuparp Lekhakula

**Affiliations:** ^1^Department of Pathology, Faculty of Medicine, Prince of Songkla University, Hat Yai, Songkhla 90110, Thailand; ^2^Department of Medicine, Faculty of Medicine, Prince of Songkla University, Hat Yai, Songkhla 90110, Thailand

## Abstract

This study was aimed to assess the clinical significances of the serum VEGF and bFGF in Thai patients with *de novo* NHL. Serum VEGF and bFGF concentrations were measured from 79 adult patients with newly diagnosed stage 2–4 non-Hodgkin lymphomas by quantitative sandwich enzyme immunoassay. At the time of diagnosis, the serum VEGF concentrations from 79 patients ranged from 72.0 to 2919.4 pg/mL, with a mean of 668.0 pg/dL. The serum bFGF concentrations ranged from undetectable to 2919.4 pg/mL, with a mean of 12.15 pg/dL. Multivariate analysis identified higher than the mean of serum VEGF, B symptoms, bulky diseases, anemia, and treatment with CHOP or R-CHOP as independent variables influencing the complete remission rate. From a Cox proportional hazards model, variables independently associated with overall survival were bone marrow involvement, more extranodal involvement, poor performance status, anemia, and higher than the mean of serum bFGF.

## 1. Introduction

Angiogenesis, the formation of new blood vessels, is of great importance in neoplastic growth and progression in both solid and hematologic malignancies. The growing of new capillaries is activated by proangiogenic molecules such as vascular endothelial growth factor (VEGF) and basic fibroblast growth factor (bFGF). The VEGF is a soluble 46-kD protein and the bFGF is an 18- to 24-kD polypeptide [1–3]. In solid tumors, the progenetic molecules act as inducers of neovascularization, thereby enhancing tumor growth and metastatic potential [4]. 

In hematologic malignancies, roles of angiogenesis were established in multiple myeloma (MM). Various studies had shown that increased microvascular density in bone marrow was associated with poor prognosis [5, 6]. Importantly, yet other studies found that antiangiogenic agents such as thalidomide or immunomodulatory drugs were associated with survival advantages in patients with MM [7–9]. 

Though predictive values of serum angiogenic factors in non-Hodgkin lymphoma (NHL) have been studied, confirmation studies in different ethnic groups should be conducted. Therefore, this study was undertaken to assess the clinical significance of the serum proangiogenic molecules, VEGF, and bFGF, in Thai patients with *de novo* NHL. 

## 2. Materials and Methods

A total of 79 adult patients with newly diagnosed stage 2–4 non-Hodgkin lymphomas was enrolled at Songklanagarind Hospital, the major tertiary care center in southern Thailand, between December 30, 2005 and April 9, 2009. Patients with a reactive test for human immunodeficiency virus or primary extranodal lymphomas were excluded. Histological classification was in accordance with the WHO classification system. Monoclonal antibodies targeting CD3, CD5, CD20, and CD79a (Dako, Glostrup, Denmark) were used for the T- or B-lineage determination. This study was approved by the Ethics Committee of Prince of Songkla University.

Clinical staging was evaluated according to the Ann Arbor staging system. Prognostic assessment was performed based on the International Prognostic Index (IPI). 

All patients were treated with a standard CHOP regimen including a minimum of six courses of cyclophosphamide, doxorubicin, vincristine, and prednisolone. Rituximab was not routinely administered in Thailand. Treatment response was classified as complete remission (CR), undetermined complete remission (CRu), partial remission (PR), stable disease (SD), or progressive disease (PD) according to the standard criteria. 

Ten mL of peripheral venous blood samples was collected from all participants before their treatment was begun. All samples were centrifuged at 2000 g for 10 minutes and frozen at −20°C soon after collection. The samples were thawed and analysed after 12–24 months' storage. Serum VEGF and bFGF concentrations were measured by quantitative sandwich enzyme immunoassay technique (Quantikine R; R&D systems, Minneapolis, MN) following the manufacturers' instructions. All analyses and calibrations were performed in duplicate. A set of standard wells containing known quantities of recombinant human VEGF and bFGF were included in all experiments. Concentrations were recorded as the mean of duplicate measurements in picograms per milliliter. The intra- and interassay variations were within the ranges given by the manufacturers.

### 2.1. Statistical Analysis

Frequency tables of baseline characteristics were analyzed with the Chi-square or Fisher's exact test. A logistic regression model was used to predict complete remission (CR). Univariate analysis of survival was performed with the Kaplan-Meier method. Overall survival (OS) was calculated as the time interval from the date of diagnosis to death or last followup. Kaplan-Meier methods were used to estimate time-to-event endpoints. Survival data between subgroups were compared using the log rank test. Multivariate analysis of OS was performed using a Cox regression model with backward elimination. Critical *P* values for entry and removal were 0.2 and 0.4, respectively. To test the main hypothesis, we forced the serum level of VEGF and bFGF into the model. Hazard ratios (HR), 95% confidence intervals (95% CI), and *P* value were obtained from the best-fit model. All the statistical analyses were performed using the R program with epicalc package. A significance level of 0.05 was used throughout all statistical tests in the study.

## 3. Results

### 3.1. Patient Characteristics and Histological Subtypes

The clinical characteristics of the 79 patients are shown in Table 1. There were 38 males and 41 females with a mean age of 55.2 (range 16–82) years. Regarding immunohistochemistry, the B-cell phenotype was shown in 69 specimens (87.3%), and 10 tissue specimens (12.7%) expressed the T-cell phenotype.

### 3.2. Serum VEGF and bFGF at the Time of Diagnosis

At the time of diagnosis, the serum VEGF concentrations from the 79 patients ranged from 72.0 to 2919.4 pg/mL with a mean of 668.0 pg/dL, median of 516.0 pg/mL, and the third quartile of 835.5 pg/mL. The serum bFGF concentration ranged from undetectable to 2919.4 pg/mL with a mean of 12.15 pg/dL, median of 9.85 pg/mL, and third quartile of 17.60 pg/mL. Associations between serum VEGF and bFGF and clinical features at diagnosis were analyzed using mean, median, and the third quartile of both angiogenetic factors as a cut-off value. No significant associations were found (data not shown). 

### 3.3. Prediction of Response Rate by Serum VEGF and bFGF

From a univariate analyses, the higher levels of serum VEGF and bFGF using the mean, median, and third quartile of both angiogenetic factors as cut-off values were not associated with a poorer complete remission (CR) rate. However, patients with B symptoms, bulky diseases, anemia, poorer performance status, high serum LDH, and T-cell immunophenotype had lower CR rates. Considering the chemotherapy regimens, CHOP and R-CHOP showed higher CR rates. Multivariate analysis identified higher than the mean of serum VEGF, B symptoms, bulky diseases, anemia, and treatment with CHOP or R-CHOP as independent variables influencing CR rate (Table 2). 

### 3.4. Prediction of Survival Rate by Serum VEGF and bFGF

**Figure 1:**
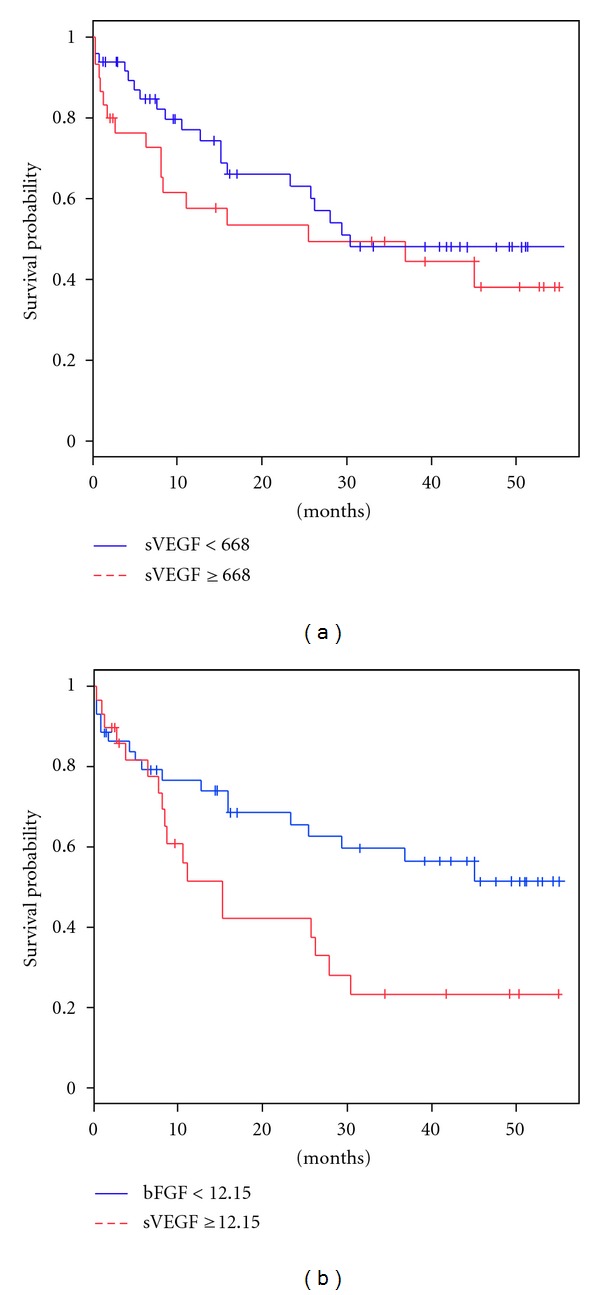
(a) Overall survival by mean VEGF (b) Overall survival by mean bFGF.

The median follow-up time was 15.1 (range 0.3–55.2) months. In univariate survival analyses, there was no association between serum VEGF and OS (Figure 1(a)).

 However, there was a significant association between shorter OS and B symptoms, more extranodal involvements, poor performance status, anemia, high serum LDH, bFGF (Figure 1(b)), and IPI. In contrast, the CHOP or R-CHOP regimens were predictors for better OS. From a Cox proportional hazards model, variables independently associated with OS were BM involvement, more extranodal involvement, poor performance status, anemia, and higher than the mean of serum bFGF as shown in Table 2.

## 4. Discussion

In this study, a high pretreatment level of serum VEGF and bFGF were independently associated with poorer CR and OS rates, respectively. Although the role of neovascularization in hematologic malignancies has been extensively explored, few studies have been conducted on the role of angiogenesis in lymphomas [10]. In addition, the predictive value of angiogenesis markers in lymphomas is still controversial due to disease heterogeneity and various detection methods [11]. However, serum proangiogenetic markers are a simple method and have the considerable advantage of not requiring an experienced pathologist to reliably assess. There have been only a few published studies on these serum proangiogenetic markers in clinical settings. However, confounding variables of known clinical prognostic factors were not considered in some of these reports. Therefore, we conducted this study to determine the independent association between these markers and clinical outcomes.

In earlier studies, Salven et al. [12–14] and Bertolini et al. [15] demonstrated a significant association between these markers and the outcomes of patients with NHL in concordance with our study. The correlation between high VEGF levels and poor CR rate also supported the hypothesis that high VEGF is responsible for an abnormal vessel structure of tumors leading to lowering drug delivery [4]. However, our more recent study found higher levels of pretreatment VEGF and bFGF, probably because the patients in our study had more advanced stages of disease. Ribatti et al. [16] and Crivellato et al. [17] also found that neovascularization was found frequently in high-grade lymphoma. The different serum VEGF and bFGF levels before treatment in patients with different degrees of disease, or due to other factors, may lead to difficulty in obtaining a single cut-off value for a predictor in all patients with NHL. In addition, the level of bFGF may be elevated due to other conditions associated with increased endothelial activity, infection, or inflammation [18, 19].

Importance roles of angiogenesis in lymphomas have been demonstrated in clinical studies. Tzankov and colleagues [20] performed immunohistochemical and morphometric studies in B-cell lymphomas and found higher microvessel density, and VEGF and COX2 in aggressive lymphomas. This result is in concordance with Ganjoo et al. [21] who reported that patients with negative stained VEGF-A or VEGF-R1 had a superior survival rate. These studies confirmed the potential importance of increased angiogenesis in prognosis and tracking of disease progression in non-Hodgkin lymphomas. 

In conclusion, our study suggests that serum VEGF and bFGF are associated with poor prognosis in patients with *de novo *non-Hodgkin lymphomas. Further studies are needed to determine more clearly whether monitoring of consecutive levels of these molecules during or after therapy could predict CR or relapse. In addition, these markers may play an important role in patient selection for antiangiogenetic treatment.

## Figures and Tables

**Table 1:** Initial characteristics of 79 patients with *de novo* NHL.

Characteristic	No (%)
Performance status (ECOG ≥2)	15 (19.0)
B symptoms	46 (58.2)
Ann Arbor stage	
I	7 (8.9)
II	22 (27.8)
III	10 (12.7)
IV	40 (50.6)
Bulky disease	28 (35.4)
Bone marrow involvement	26 (32.9)
High serum LDH	46 (58.2)
Histological subtype	
Diffuse large cell lymphoma	54 (68.4)
Follicular lymphoma	8 (10.1)
Mantle cell lymphoma	3 (3.8)
Lymphoblastic lymphoma	2 (2.5)
Burkitt's lymphoma	1 (1.3)
Mucosa-associated lymphoid tissue	1 (1.3)
Peripheral T-cell lymphoma, unclassified	6 (7.6)
Anaplastic large cell lymphoma	3 (3.8)
Angioimmunoblastic T-cell lymphoma	1 (1.3)
International prognostic index	
Low risk	24 (30.4)
Low-intermediate risk	20 (25.3)
High-intermediate risk	21 (26.6)
High risk	14 (17.7)
Chemotherapy	
CHOP	44 (55.7)
R-CHOP	22 (27.8)
Other	7 (8.9)
No chemotherapy	6 (7.6)

**Table 2:** Univariate and multivariate analysis for complete response and overall survival.

	Complete response		Overall survival	
Variable	Univariate OR (95% CI)^#^	Multivariate OR (95% CI)^#^	Univariate HR (95% CI)^#^	Multivariate HR (95% CI)^#^
Age: ≥60 years	1.34 (0.52, 3.45)	Not tested	1.31 (0.68, 2.54)	Not tested
Sex: female	0.94 (0.37, 2.39)	Not tested	1.21 (0.62, 2.33)	Not tested
B symptoms	10.29 (3.05, 34.71)***	10.6 (1.79, 62.65)**	3.20 (1.53, 6.71)**	Not tested
Bulky diseases	2.95 (1.1, 7.88)*	5.32 (1, 28.23)*	0.84 (0.42, 1.68)	Not tested
Stage: III-IV	2.1 (0.77, 5.73)	Not in final model	1.94 (0.93, 4.05)	Not in final model
BM involvement	1.89 (0.69, 5.2)	Not in final model	1.54 (0.79, 3.0)	0.30 (0.11, 0.82)*
Liver involvement	1.27 (0.41, 3.91)	Not tested	1.50 (0.74, 3.01)	Not in final model
Extranodal involvement: >2	2.09 (0.57, 7.6)	Not in final model	2.47 (1.19, 5.14)*	2.60 (1.03, 6.51)*
Performance status: ECOG 2–4	6.3 (1.54, 25.83)*	Not in final model	4.37 (2.12, 8.98)***	4.44 (1.84, 10.72)***
High serum LDH	3.43 (1.22, 9.67)*	Not in final model	3.85 (1.67, 8.89)**	1.53 (0.53, 4.46)
IHC: T-cell	4.45 (1.05, 18.94)*	Not in final model	2.22 (0.92, 5.36)	Not in final model
Anemia	6.31 (2.14, 18.63)***	10.9 (1.63, 73)**	3.92 (1.83, 8.42)***	4.14 (1.40, 12.26)*
Lymphopenia	2.18 (0.8, 5.96)	Not in final model	1.80 (0.90, 3.57)	2.25 (1.00, 5.07)*
IPI: HI-high	1.94 (0.53, 7.13)	Not in final model	3.24 (1.60, 6.53)**	Not tested
Chemotherapy:				
CHOP	0.25 (0.05, 1.4)*	0.2 (0.02, 2.42)***	0.41 (0.18, 0.90)*	0.13 (0.04, 0.41)***
R-CHOP	0.07 (0.01, 0.51)	0.01 (0, 0.26)	0.24 (0.09, 0.64)**	0.04 (0.01, 0.19)***
Serum VEGF (pg/mL)				
>668.0 (mean)	2 (0.75, 5.3)	5.33 (0.97, 29.27)*	1.37 (0.71, 2.64)	Not in final model
>516.0 (median)	2.12 (0.82, 5.49)	Not tested	0.99 (0.51, 1.90)	Not tested
>835.5 (3rd Qu)	1.76 (0.58, 5.38)	Not tested	1.03 (0.48, 2.18)	Not tested
Serum bFGF (pg/mL)				
>12.15 (mean)	1.19 (0.44, 3.23)	1.68 (0.37, 7.71)	2.08 (1.07, 4.03)*	3.31 (1.32, 8.30)*
>9.85 (median)	1.86 (0.69, 4.98)	Not tested	1.75 (0.90, 3.42)	Not tested
>17.60 (3rd Qu)	1.24 (0.4, 3.87)	Not tested	1.96 (0.97, 3.94)	Not tested

^#^Significance level: ***0.001, **0.01, *0.05.
